# Genetic and Ecologic Variability among *Anaplasma phagocytophilum* Strains, Northern Italy

**DOI:** 10.3201/eid2006.131023

**Published:** 2014-06

**Authors:** Ivana Baráková, Markéta Derdáková, Giovanna Carpi, Fausta Rosso, Margherita Collini, Valentina Tagliapietra, Claudio Ramponi, Heidi C. Hauffe, Annapaola Rizzoli

**Affiliations:** Fondazione Edmund Mach, Trento, Italy (I. Baráková, Giovanna Carpi, F. Rosso, M. Collini, V. Tagliapietra, H.C. Hauffe, A. Rizzoli);; Institute of Zoology, Slovak Academy of Sciences, Bratislava, Slovak Republic (I. Baráková, M. Derdáková);; Institute of Parisitology SAS, Košice, Slovak Republic (M. Derdáková);; Yale School of Public Health, New Haven, CT, USA (G. Carpi);; Ospedale Santa Chiara, Trento (C. Ramponi)

**Keywords:** *Ixodes ricinus*, bacteria, vector-borne infections, tick-borne, *Anaplasma phagocytophilum*, human granulocytic anaplasmosis, *groEL*, *msp4*, Valle dei Laghi, Italy

**To the Editor:** The tick-borne pathogen *Anaplasma phagocytophilum* is an increasing potential public health threat across Europe. Its intraspecific genetic variability is associated with different reservoir host and vector tick species (*1*–*4*); however, the roles of various vertebrates as competent reservoirs of *A. phagocytophilum* in Europe need clarification (*1*). During March 2011–June 2013, we studied the prevalence and genetic variability of *A. phagocytophilum* in 821 questing *Ixodes ricinus* ticks (155 adults [A], 666 nymphs [N] collected by standard blanket dragging) and 284 engorged ixodid ticks (61A, 191N, 21 larvae [L]) collected from humans, dogs, sheep, hunted wild ungulates, live-trapped birds, and rodents. Blood samples from 1,295 rodents (yellow-necked mice [*Apodemus flavicollis*]), bank voles [*Myodes glareolus*], and harvest mice [*Moscardinus avellanarius*]) were also analyzed. All animal-handling procedures and ethical issues were approved by the Provincial Wildlife Management Committee (authorization n. 595 issued on 04.05.2011). The study site, Valle dei Laghi (northeastern Italian Alps), is a well-studied focus of emerging tick-borne pathogens in northern Italy (*4*).

Tick species were identified morphologically and by molecular analyses by using 16SrRNA sequences. *A. phagocytophilum* was detected in questing and feeding *I. ricinus* ticks by using a nested PCR amplification of the partial 16S rRNA gene (546-bp fragment) as described (*4*,*5*) and in rodent blood by using a real time-PCR assay targeting the *msp2* gene (77 bp) (*6*). All positive samples were confirmed by using Sanger sequencing.

Overall prevalence of *A. phagocytophilum* in questing *I. ricinus* ticks was 1.8% (6A, 9N of 821) (Table). Among engorged ticks, only *I. ricinus* ticks were found positive for *A. phagocytophilum*, although tick species such as *I. hexagounus* (20 ticks from dogs and birds), *I. trianguliceps* (11 from rodents), and *I. turdus* (1 from a bird) were also analyzed. Infection prevalence in ticks from various hosts was: 4.3% (5N/115) in ticks from humans, 9.1% (1N/30) in ticks from dogs, 14.3% (4A, 1N, 2L/49) in ticks from wild ungulates, 7.7% (1A/30) in ticks from sheep, 10.7% (3N/28) in ticks from birds, and 6.1% (3N/49) in ticks from rodents (Table). Prevalence in rodent blood samples (*A. flavicolis* mice, *M. avellanarius* mice, *M. glareolus* bank voles) was 0.3% (4/1,295); only bank voles had positive results. None of the feeding *I. ricinus* larvae collected from rodents were infected with *A. phagocytophilum*.

**Table:** Prevalence of *Anaplasma phagocytophilum* in questing and feeding *Ixodes* ticks from Valle dei Laghi, northern Italy*

Sample source	Tick host group/no. sampled per group	No. ticks per species				No. ticks per stage (A/N/L)†	No. ticks screened	No. *A. phagocytophilum-*infected ticks (% positive* I. ricinus*/% positive total ticks)‡
		*I. ricinus*	*I. hexagonus*	*I. trianguliceps*	*I. turdus*			
Questing ticks		821	0	0	0	155/666/0	821	15 (1.8/1.8)
Feeding ticks	Humans/111	115	0	0	0	32/83/0	115	5 (4.3/4.3)
	Dogs/17	11	19	0	0	5/23/2	30	1 (9.1/3.3)
	Wild ungulates/11	49	0	0	0	16/28/3	49	7 (14.3/14.3)
	Sheep/13	13	0	0	0	13/0/0	13	1 (7.7/7.7)
	Wild rodents/44	38	0	11	0	4/29/16	49	3 (7.9/6.1)
	Wild birds/28	26	1	0	1	0/27/1	28	3 (11.5/10.7)

*Feeding ticks were removed from humans, domestic dogs (*Canis familiaris*), wild ungulates (roe deer *Capreolus capreolus,* red deer *Cervus elaphus*), sheep (*Ovis aries*), wild rodents (yellow-necked mouse *Apodemus flavicollis*, harvest mouse *Moscardinus avellanarius*, bank vole *Myodes glareolus*), and wild birds (robin *Erithacus rubecula*, blackbird *Turdus merula*, thrush *Turdus philomelos*, great tit *Parus major*, nightingale *Luscinia megarhynchos*, and jay *Garrulus glandarius*). †A, adult; N, nymph; L, larva. ‡Only *I. ricinus* ticks were found to be *A. phagocytophilum*–infected.

We amplified and sequenced 2 genetic loci, *groEL* and *msp4*, from samples that were positive for *A. phagocytophilum*, which are known to be useful for phylogenetic studies (*4*,*7*,*8*). MrBayes v3.1.2 (http://sourceforge.net/projects/mrbayes/files/mrbayes/3.2.1/ ) was used to construct Bayesian phylogenetic trees for each gene (*9*). We deposited 54 new *A. phagocytophilum* sequences in GenBank with accession numbers KF031380–KF031433. Fourteen and 9 unique *groEL* and *msp4 A. phagocytophilum* genotypes, respectively, were found to circulate in this alpine valley.

The phylogenetic trees for *groEL* (Technical Appendix Figure) and *msp4* (Technical Appendix Figure, panel B) loci have similar topologies with strong support for 2 main clades (Technical Appendix Figure, panels A and B), each with different host and vector association. The first clade (clade 1) contained sequences from questing *I. ricinus* ticks and engorged ticks collected from humans, dogs, wild ungulates, rodents, sheep, and birds. Our findings suggest that humans are exposed to several *A. phagocytophilum* genotypes exclusively from clade 1 (Technical Appendix Figure, panels A and B). Our 3 unique *A. phagocytophilum* sequences were from 3 *I. ricinus* nymphs that fed on the same human clustered within this clade, but no clinical symptoms were observed.

The second clade (clade 2) includes sequences from rodents, specifically, bank voles (*M. glareolus*), other voles and shrews. Among tick species we found *I. persulcatus* to belong to this clade (Technical Appendix Figure, panels A and B). We have found no evidence of circulation of this genotype in other hosts or in questing or engorged *I. ricinus* ticks in previously published data or in this study (Technical Appendix Figure, panels A and B, clade 2). This finding suggests that the *A. phagocytophilum* genotype associated with mice, voles, and shrews in Europe may be maintained in enzootic cycles by another tick vector, such as *I. trianguliceps*, as observed in the UK for the field vole (*Microtus agrestis*) (*8*). This so-called ecologic strain probably does not represent an immediate threat to humans in northern Italy, unlike the rodent strain reported in the USA, since it occurs in very low prevalence, and because *I. trianguliceps* is an endophilic tick species that is unlikely to come into contact with humans.

In 1 questing *I. ricinus* tick at the nymphal stage, we detected a *groEL* sequence (KF031399) identical to a sequence isolated from humans with human granulocytic anaplasmosis in Europe (AF033101). The *msp4* sequence for the same sample (KF031406) belonged to clade 1, and contained sequences of a strain found in 96 infected persons in the United States. This suggests that *>*1 human pathogenic strain now circulates in the investigated area. However, we did not find this strain in any of the host-fed ticks analyzed, so the host responsible for maintaining the circulation of this pathogenic strain must be identified before any recommendation for preventive measures can be provided.

Technical Appendix Baysesian analysis of of *Anasplasma phagocytophilum* *groEL* gene partial sequences and 73 *msp4* gene partial sequences.
