# Supplementary material for: Genetic and Ecologic Variability among Anaplasma phagocytophilum Strains, Northern Italy
Source: Emerg Infect Dis. 2014 Jun;20(6):1082–5. doi: 10.3201/eid2006.131023 (PMC4036757; doi:10.3201/eid2006.131023)
Supplement: Technical Appendix — Baysesian analysis of of Anasplasma phagocytophilum groEL gene partial sequences and 73 msp4 gene partial sequences. [file 13-1023-Techapp-s1.pdf]

Article DOI: 10.3201/eid2006.131023

Genetic and Ecologic Variability among *Anaplasma phagocytophilum* Strains, Northern Italy

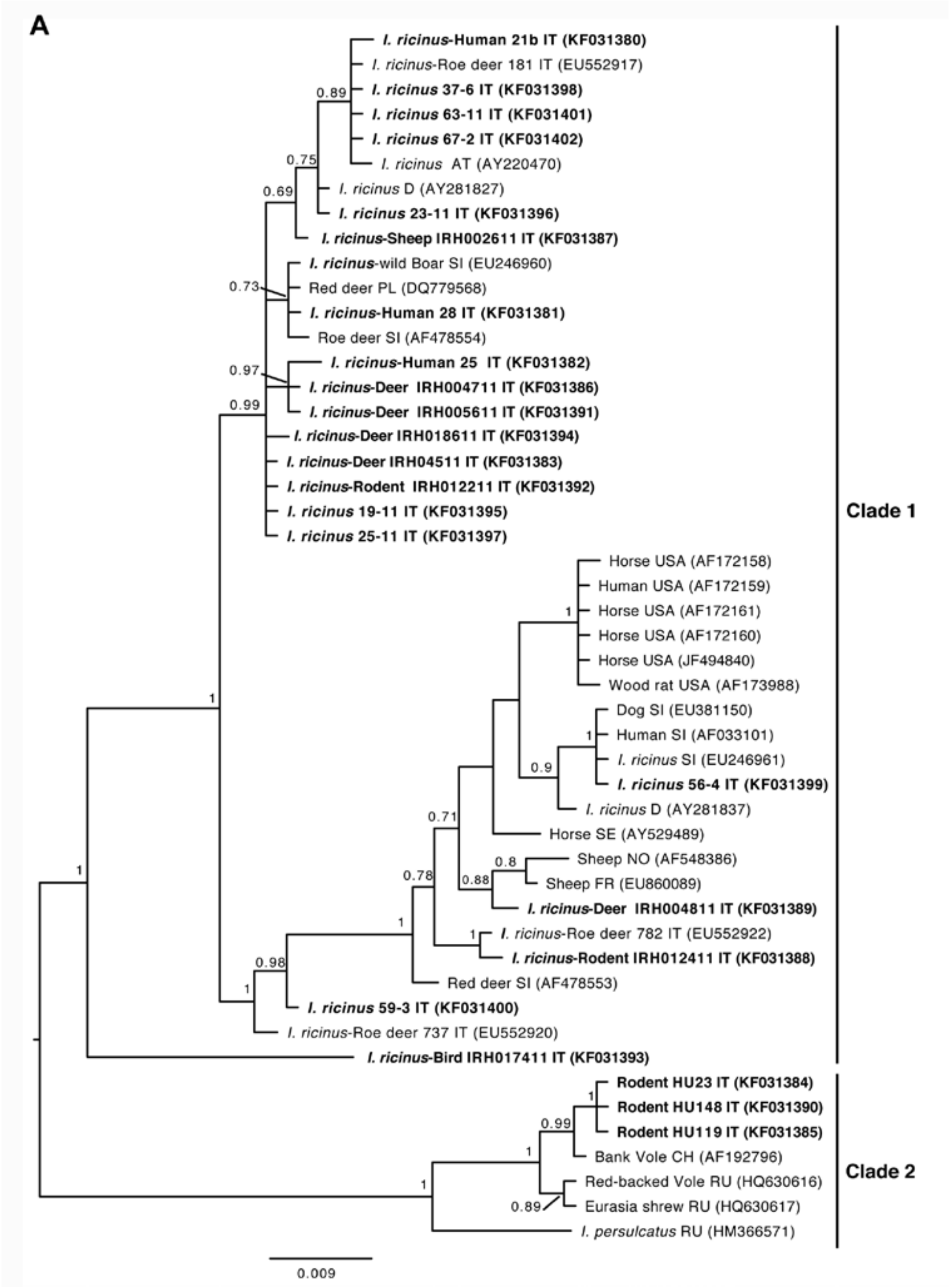

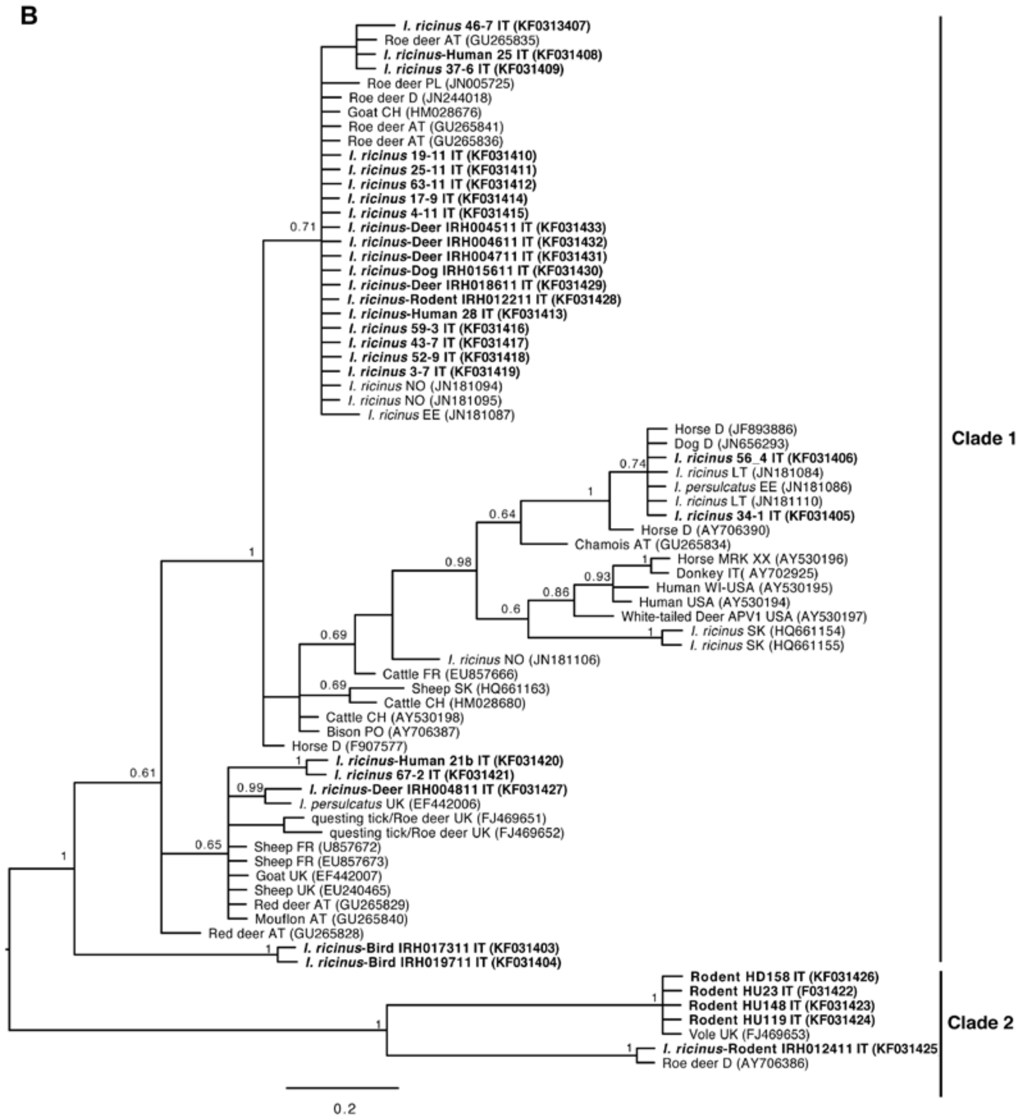

Technical Appendix Figure. A) 50% majority rule consensus phylogenetic trees constructed by using Bayesian analysis of *Anasplasma phagocytophilum groEL* gene partial sequences (1,119 bp); B) 73 *msp4* gene partial sequences (300 bp). Markov chains were run for 1,000,000 generations. The first 1,000 trees were discarded and the remaining trees were used to construct the tree. Posterior probabilities of >0.60 are indicated above branches. New sequences are shown in boldface. Each *A. phagocytophilum* sequence is indicated with source: questing tick (e.g., *I. ricinus*), engorged tick (e.g., *I. ricinus*-Human), or host blood (e.g., red deer), international organization for standardization α-2 country codes and GenBank accession numbers are in parentheses. Scale bars indicate nucleotide substitutions per site.
